# Dual-Source Photon-Counting Computed Tomography—Part I: Clinical Overview of Cardiac CT and Coronary CT Angiography Applications

**DOI:** 10.3390/jcm12113627

**Published:** 2023-05-23

**Authors:** Filippo Cademartiri, Antonella Meloni, Laura Pistoia, Giulia Degiorgi, Alberto Clemente, Carmelo De Gori, Vincenzo Positano, Simona Celi, Sergio Berti, Michele Emdin, Daniele Panetta, Luca Menichetti, Bruna Punzo, Carlo Cavaliere, Eduardo Bossone, Luca Saba, Riccardo Cau, Ludovico La Grutta, Erica Maffei

**Affiliations:** 1 Department of Radiology, Fondazione Monasterio/CNR, 56124 Pisa, Italy; antonella.meloni@ftgm.it (A.M.); laura.pistoia@ftgm.it (L.P.); giuliadegiorgi995@gmail.com (G.D.); clemente@ftgm.it (A.C.); degoricarmelo87@gmail.com (C.D.G.); positano@ftgm.it (V.P.); emaffei@ftgm.it (E.M.); 2 Department of Bioengineering, Fondazione Monasterio/CNR, 56124 Pisa, Italy; 3 BioCardioLab, Department of Bioengineering, Fondazione Monasterio/CNR, 54100 Massa, Italy; simona.celi@ftgm.it; 4 Cardiology Unit, Ospedale del Cuore, Fondazione Monasterio/CNR, 54100 Massa, Italy; sergio.berti@ftgm.it; 5 Department of Cardiology, Fondazione Monasterio/CNR, 56124 Pisa, Italy; emdin@ftgm.it; 6 Institute of Clinical Physiology, National Council of Research, 56124 Pisa, Italy; daniele.panetta@ifc.cnr.it (D.P.); luca.menichetti@ifc.cnr.it (L.M.); 7 Department of Radiology, IRCCS SynLab-SDN, 80131 Naples, Italy; bpunzo@sdn-napoli.it (B.P.); carlo.cavaliere@synlab.it (C.C.); 8 Department of Cardiology, Ospedale Cardarelli, 80131 Naples, Italy; ebossone@hotmail.com; 9 Department of Radiology, University Hospital, 09042 Monserrato, Italy; lucasabamd@gmail.com (L.S.); riccardocau00@gmail.com (R.C.); 10 Department of Radiology, University Hospital “P. Giaccone”, 90127 Palermo, Italy; lagruttaludovico@gmail.com

**Keywords:** photon-counting computed tomography, computed tomography angiography, coronary computed tomography, cardiac computed tomography, photon-counting detector

## Abstract

The photon-counting detector (PCD) is a new computed tomography detector technology (photon-counting computed tomography, PCCT) that provides substantial benefits for cardiac and coronary artery imaging. Compared with conventional CT, PCCT has multi-energy capability, increased spatial resolution and soft tissue contrast with near-null electronic noise, reduced radiation exposure, and optimization of the use of contrast agents. This new technology promises to overcome several limitations of traditional cardiac and coronary CT angiography (CCT/CCTA) including reduction in blooming artifacts in heavy calcified coronary plaques or beam-hardening artifacts in patients with coronary stents, and a more precise assessment of the degree of stenosis and plaque characteristic thanks to its better spatial resolution. Another potential application of PCCT is the use of a double-contrast agent to characterize myocardial tissue. In this current overview of the existing PCCT literature, we describe the strengths, limitations, recent applications, and promising developments of employing PCCT technology in CCT.

## 1. Introduction

Non-invasive cardiac imaging is a constantly and rapidly evolving field of medicine that has recently experienced major technological breakthroughs. The diagnostic need is growing in several applications of major imaging technologies, and is mainly driven by ruling in or out significant coronary luminal stenosis or coronary anomalies, investigating myocardial tissue characteristics, assessing coronary atherosclerotic plaque morphology and characteristics, and providing adequate pre-operative support for interventional procedures [1,2,3,4,5,6]. Computed tomography (CT) represents a powerful tool to address these clinical indications and it can already be considered the primary diagnostic tool in cardiovascular medicine when an angiographic/anatomical assessment is needed. However, for the heart and the coronary arteries, some limitations still remain (also depending on the technology involved), mostly related to limited contrast resolution, sub-optimal spatial resolution for angiography in patients with severe calcifications and stents, metallic/blooming/beam-hardening artifacts, and sub-optimal tissue characterization capabilities [7,8]. Moreover, there are a few direct risks associated with CT scanning, such as the increased exposure to ionizing radiation and the possibility of contrast-induced allergic reactions or nephropathy.

Photon-counting computed tomography (PCCT) is a newly introduced CT detector technology that has been developed for more than 15 years and has recently entered the clinical field. After Food and Drug Administration (FDA) clearance in September 2021, Siemens Healthineers released the world’s first commercial PCCT scanner (Naeotom Alpha.

PCCT is based on a completely new generation of X-ray detectors [9,10,11,12]. Until now, the standard technology for CT detectors was energy-integrating detectors (EIDs) in which photons hit the detector surface, and the detected signal is proportional to the total energy deposited by all photons without specific information about an individual photon or its energy; this is because photons are converted into light with several inherent limitations, such as limited spatial resolution, scattering, loss of low energy photons, and no energy level discrimination. PCCT detectors, instead, are composed of semiconductor detector materials made of cadmium telluride, cadmium zinc telluride, or silicon that directly convert each X-ray photon into electron hole pairs [9,13,14]. This can result in the direct detection of photons, enabling them to be counted (i.e., photon counting) and to be separated into their specific energy levels, while eliminating noise at the electronic level. This detector characteristic has major clinical benefits in cardiovascular imaging by more than doubling spatial resolution, reducing electronic noise and artifacts, decreasing the X-ray dose and amount of contrast media, and allowing simultaneous multi-energy acquisition to characterize myocardial tissue and/or atherosclerotic coronary plaques [7,9,11,12,15,16,17].

In this current overview of the existing PCCT literature, we describe the strengths, limitations, recent applications, and promising developments of employing PCCT technology in CCT.

## 2. Basic Features and Technical Improvements Related to PCCT Technology

PCCT can operate in two different modalities: conventional and spectral. The first corresponds to single-energy CT, in which X-ray photons are not differentiated by their energy but are only summed. Spectral data can be used through two distinct mechanisms: the weighting of energy and the decomposition of the material.

With the first approach, PCCT provides improved noise performance in comparison with conventional state-of-the-art CT scanners. Indeed, it is possible to assign custom weights to specific energy bins in order to improve image quality. This enables normalization of the weight of low-energy X-ray photons and leads to an improvement in the contrast-to-noise ratio between soft tissues [18] or a correction in beam-hardening artifacts [8,19].

Decomposition of the material spectral analysis, instead, provides information about the distribution of certain elements within the image (e.g., iodine, calcium, and gadolinium) by measuring the energy-dependent material-specific X-ray attenuation. This PCCT technology also enables the generation of virtual mono-energetic images [8,18]. In contrast to dual-energy computed tomography (DECT), PCCT acquires data in three or more energy regimes, enabling more than two different contrast agents to be discerned and quantification with a K-edge (e.g., gold and gadolinium) in the diagnostic energy range [8,20,21,22]. This energy threshold capability also allows PCCT to improve the contrast-to-noise ratio and the capability of CT to generate separate quantitative maps for each component [8,18].

Moreover, the detector element size in PCCT is smaller than that in EID. Since PCCT detectors convert X-ray photons directly into electronical charges without scintillator layers, the spatial resolution in PCCT is not limited by the need to separate proximate detector elements with reflecting layers to minimize crosstalk between neighboring detector elements and prevent image degradation [18]. This technology enables higher spatial resolution in comparison with conventional CT [18].

**Figure 1:**
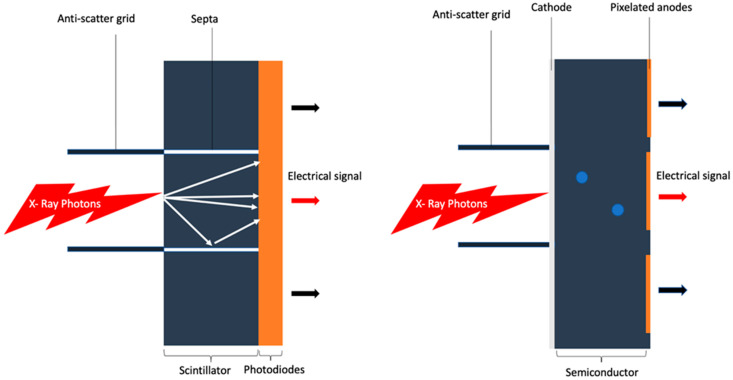
Schematic representation of EIDs vs. PCDs. Schematic representation of energy integrating detector system (on the left): The X-ray photons are absorbed by the scintillator and converted into visible light which is then collected by photo diodes that generate an electrical signal. Schematic representation of photon counting detector system (on the right): The X-ray photons are absorbed in a semiconductor detector material that directly converts each X-ray photon into an electron hole.

A schematic representation of EIDs and PCDs is shown in Figure 1.

## 3. Clinical Implications of Photon-Counting CT Technology

The main clinical translation of new PCCT technology in the cardiac field application is related to the higher spatial resolution and to the multiparametric/multi-energetic nature of the information collected. 

The presence of semiconductor materials and the detector element size result in higher spatial resolution [8,14,18]. This provides a much more detailed capability of visualization of smaller anatomical structures with more contrast resolution, including coronary lumen and stent patency. Improved spatial resolution can be also helpful for a better evaluation of high-risk plaque features, namely low-attenuation plaque, spotty calcifications, positive remodeling, and napkin-ring sign [18,23]. PCCT technology enables the counting of both the cumulative number of photons and their energy distribution, leading to improved contrast-to-noise ratios and energy discrimination capabilities. This translates into increased iodine contrast, reduced administered radiation dose, or reduced contrast media volume required to obtain comparable enhancement [24,25,26]. Finally, multi-energy capabilities of PCCT detectors could be useful to reduce metal artifact, differentiate components of coronary atherosclerosis plaque, and discern between different exogenous contrast agents [18,24,27,28,29].

Table 1 summarizes the main benefits of PCCT and effects on cardiovascular applications.

Table 2 summarizes previous studies on PCCT in cardiovascular imaging.

### 3.1. Coronary Lumen

The PCCT system provides clinical benefits in the luminal assessment of coronary arteries, enabling improved spatial resolution and contrast-to-noise ratio [25].

In a recent prospective study of 14 patients who underwent both PCCT and conventional CT angiography, PCCT demonstrated a score improvement for overall quality and diagnostic confidence of 57% (95% CI: 41, 72) and 55% (95% CI: 39, 70), respectively, and 48% (95% CI: 33, 63), 51% (95% CI: 35, 67), and 69 (95% CI: 53, 82) for coronary proximal lumen, coronary distal lumen, and coronary wall, respectively [30]. These findings were also supported by a phantom study reporting that PCCT images had a 2.3-fold increased detectably index for coronary lumen in comparison with conventional CT images [30]. In an in vitro study, PCCT was compared with EID under various conditions of simulated patient size (small, medium, and large), demonstrating lower noise magnitude and higher noise frequency peak with better spatial resolution compared to EID [31]. Moreover, PCCT can be advantageous in quantifying luminal stenosis in heavily calcified plaques in comparison with conventional CT scanners, as demonstrated in a recent phantom study, especially for concentric heavy calcified plaque configuration [12]. Similar results were also reported by Li et al., who demonstrated a reduction in partial volume and blooming artifacts resulting in a finer stenosis assessment [41]. In parallel, PCCT enables CCT K-edge imaging using a gadolinium-based contrast agent. An ex vivo coronary artery study showed an improved luminal depiction with clear differentiation among the intravascular gadolinium-based contrast agent, calcified plaque, and stent material [21]. Similar results have also been recently described using iodinated contrast agents [42].

A different approach to assess the vessel lumen is the use of emerging image reconstruction algorithms based on spectral CT [32]. Allmendinger et al. investigated the performance of a novel calcium-removal image reconstruction algorithm (called PureLumen) to eliminate only the calcified contribution of an anthropomorphic thorax phantom attached to an artificial motion device, simulating realistic cardiac motion [32]. The authors demonstrated decreased blooming artifacts and an improvement in image interpretability [32]. PCCT enables an “always available” multi-energy discrimination, overcoming a current dilemma in cardiac CT, represented by the infeasibility of high temporal resolution and multi-energy imaging acquisition [43].

In addition, improved spatial resolution of PCCT allows a proportion of improvement in diagnostic quality for pericoronary fat tissue of 36% (95% CI: 22, 52) in comparison with conventional CT scanners [30].

**Figure 2:**
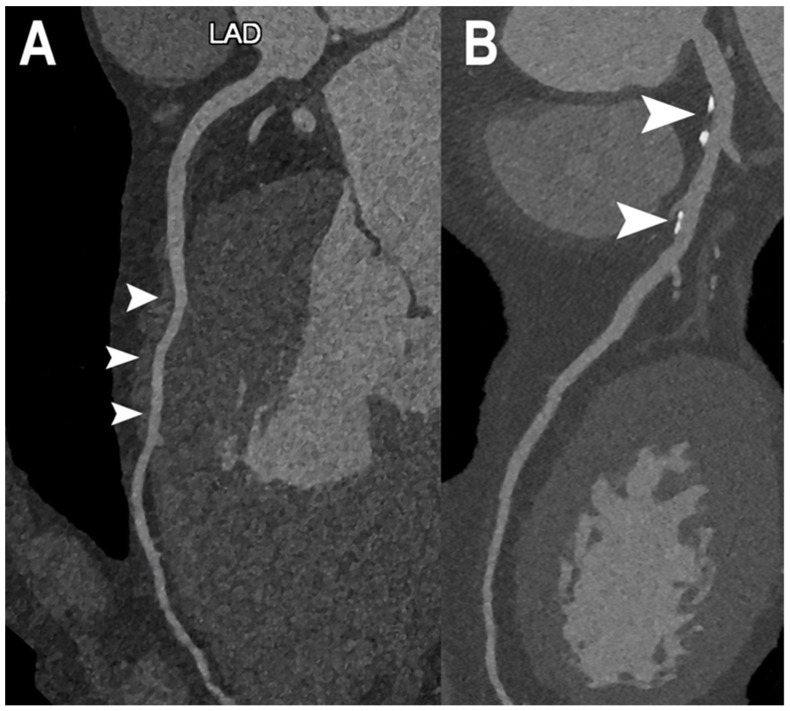
Cardiac PCCT examples of normal and non-obstructive CAD. The figure shows two examples of left anterior descending coronary artery (LAD; (**A**,**B**)). In A, the LAD is completely normal without any sign of coronary artery disease (CAD); the only finding is a deep intramyocardial course of the middle segment ((**A**); arrowheads). In (**B**), there are at least two atherosclerotic plaques ((**B**); arrowheads) with predominantly calcified phenotype, without any signs of significant obstruction of the coronary lumen. What is special about this imaging with PCCT is that this very high detail and spatial resolution is obtained with standard imaging protocols and does not require high radiation dose.

**Figure 3:**
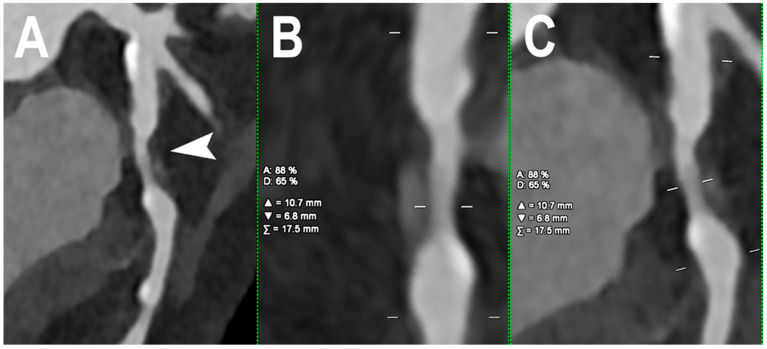
Cardiac PCCT example of obstructive CAD. The figure shows an example of left circumflex stenosis due to a predominantly non-calcified atherosclerotic plaque with non-significant positive remodeling ((**A**); arrowhead). This example is obtained in medium resolution which, in this case, is a 0.4 mm slice thickness. The quantitative assessment ((**B**,**C**); orthogonal longitudinal views of the stenosis) of the stenosis shows a 65% lumen reduction in diameter and an 88% lumen reduction in area.

**Figure 4:**
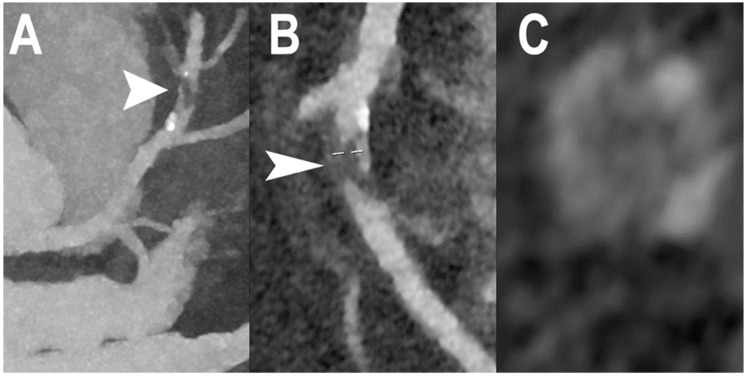
Cardiac PCCT example of obstructive CAD. The figure shows an example of high-grade coronary artery stenosis in the middle portion of left anterior descending (LAD) coronary artery (**A**–**C**). The plaque ((**A**,**B**); arrowheads) shows predominantly non calcified phenotype and low inner core density (**C**). This example is obtained with high resolution mode (100 microns spatial resolution and minimum slice thickness of 0.2 mm).

Figure 2 shows PCCT images of normal and non-obstructive coronary artery disease (CAD) while PCCT examples of obstructive CAD are shown in Figure 3 and Figure 4.

### 3.2. Coronary Stent

Current state-of-the-art CT scanners do not always allow optimal assessment of the vessel lumen in patients with coronary stents due to several technical issues (e.g., metallic, blooming, and beam-hardening artifacts, as well as limited spatial resolution) [44,45].

In a recent in vitro study, PCCT was compared with conventional CT scanners in the evaluation of 18 different coronary stents. The authors reported superior in-stent visibility, and fewer blooming and partial volume artifacts, with a smaller increase in the attenuation of the lumen inside the stent for PCCT [23]. These results were also confirmed in different in vitro studies [34,46].

Recently, several in-human studies investigated the advantages of PCCT in coronary stent evaluation [30,33]. Boccalini et al. compared the image quality of in vivo coronary stents between PCCT and conventional CT, reporting a superior stent and lumen visibility with fewer artifacts and lower dose radiation (25.7 mGy for PCCT vs. 35.7 mGy for conventional CT, *p* = 0.02) [33]. Similar results were also reported by Si-Mohamed et al., who compared the quality of CCT scans obtained with PCCT technology and conventional CT scanners [30]. The authors described a proportion of improvement with PCCT images for coronary stent of 92% (95% CI: 71, 98) in diagnostic quality with a lower mean dose-length product (411 mGy vs. 592 ± 171, *p* < 0.01) [30].

**Figure 5:**
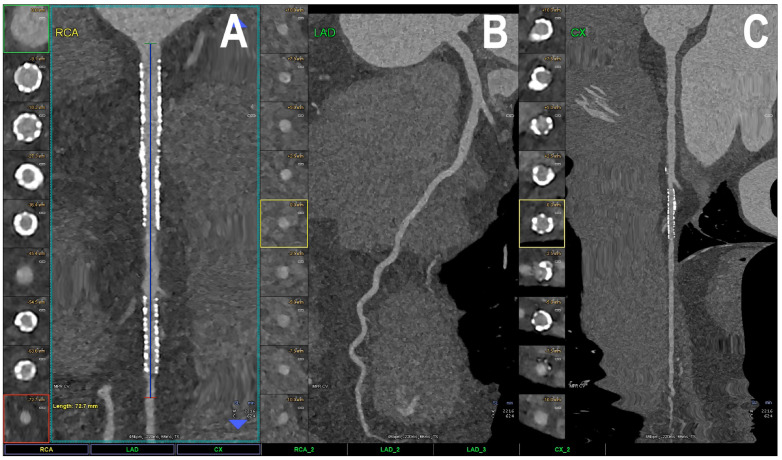
Cardiac PCCT example of a complete coronary tree with multiple stents. The figure shows the complete coronary tree of a patient with multiple stents at follow-up (**A**–**C**). There are two stents at the level of the proximal and middle right coronary artery (**A**) and one stent on the marginal branch of the left circumflex coronary artery (**C**); the left anterior descending (LAD; (**B**)) the coronary artery is normal without any detectable atherosclerotic disease. All stents are perfectly visualized in their inner struts and also in their inner lumen, which is not normal for cardiac CT without photon counting technology.

A PCCT image of a complete coronary tree with multiple stents is shown in Figure 5. 

**Figure 6:**
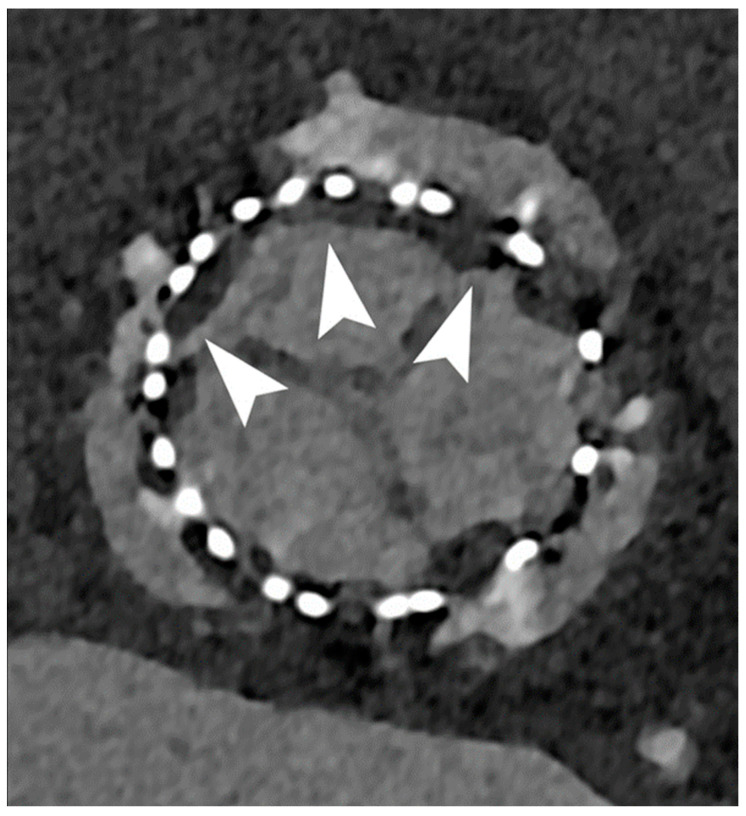
Cardiac PCCT of aortic valve prosthesis. In this example we show a follow-up of an aortic valve prosthesis that shows significant signs of Hypo-Attenuating Leaflet Thickening (HALT) which can be due to thrombotic apposition (arrowheads) and may impair valve leaflet motion. With PCCT this very thin layer of hypodense tissue can be easily seen.

This new technology can also be applied to cardiac valve and prosthetic valve complications (Figure 6), especially in patients with sub-optimal ultrasound views or with field inhomogeneities in cardiac magnetic resonance [35].

### 3.3. Coronary Artery Calcium Score

Coronary artery calcium (CAC) is generally quantified on CT using the Agatston score [6,47,48]. The factor used for the calculation of the Agatston score is selected according to fixed HU thresholds and is highly dependent on the maximum attenuation of a calcified plaque [47,48]. PCCT allows for reducing the level of electronic noise, resulting in less image noise, fewer streak artifacts, and more stable Hounsfield unit (HU) numbers [49,50]. In an in vitro study, coronary calcium scoring was compared between PCCT and CT scanners, reporting a comparable CAC score for the routine clinical protocol [36]. Further, PCCT increased detectability and accuracy in CAC with a reduced slice thickness [36]. Symons et al. investigated the performance of PCCT at standard and reduced radiation doses in a dedicated cardiac CT phantom, ten ex vivo hearts, and ten asymptomatic volunteers [38]. Phantom and in vivo human studies demonstrated the potential of PCCT to improve CAC score image quality and/or to reduce radiation dose while maintaining diagnostic image quality [38].

An excellent correlation and agreement were demonstrated between the CAC score derived from PCCT and conventional CT in 26 calcified coronary lesions from 5 cadaveric hearts [10], supporting the potential use of the Agatston score derived from PCCT in clinical practice.

Eberhard et al. investigated the accuracy of the CAC score on PCCT in comparison with conventional CT scanners and explored the optimal virtual mono-energetic images and iterative image reconstruction algorithm at different radiation doses in a phantom and patients [37]. The study showed decreasing CAC score at increasing iterative image reconstruction algorithm levels (*p* < 0.001) and increasing keV levels (*p* < 0.001) [37].

PCCT may also have substantial benefits for aortic valve calcification score imaging. In an initial report of five patients who underwent transcatheter aortic valve replacement planning CT using a novel PCCT, an excellent correlation of aortic valve calcium score and volumes between virtual non-contrast and true-contrast images (r = 0.945, *p* = 0.01; r = 0.938, *p* = 0.01; respectively) was demonstrated [35].

### 3.4. Atherosclerotic Plaque Composition

Besides stenosis assessment, a growing body of evidence has emphasized the need for a more detailed evaluation of atherosclerotic plaque morphology and characteristics [51,52,53,54,55,56]. Direct visualization of coronary plaque components (e.g., thin cap fibroatheroma, microcalcifications) is problematic in conventional CT scanners [5,57,58]. PCCT, thanks to its improved spatial resolution, provides an improvement in diagnostic quality for coronary calcification and noncalcified plaque of 100% and 45% (95% CI: 28, 63), respectively [30]. In an in vitro study, PCCT provided superior detectability for simulated 0.5 mm thick non-calcified plaques (AUC ≈ 95% vs. AUC ≈ 75%) and lipid-rich atherosclerotic plaques (AUC = 85% vs. AUC = 77.5%) in comparison with EID [31].

Further, spectral analysis also allows multi-material mapping via a material decomposition algorithm. A preliminary ex vivo study investigated the capabilities of PCCT to differentiate components of coronary atherosclerosis plaque in 23 histologically demonstrated atheromatous plaques from post-mortem human coronary arteries [9]. PCCT was demonstrated to identify plaque components by measuring differences in contrast agent concentration and spectral attenuation [9]. Another ex vivo study confirmed the significant potential of PCCT to distinguish components of vulnerable atherosclerotic plaque (calcium, iron, lipid surrogate, and cellular surrogate) based on their different photo-electric and Compton effects with good correlation with histological slices [59]. Furthermore, Jorgensen et al. demonstrated the ability of PCCT to quantify vasa vasorum density as a marker of early atherosclerosis changes in perfusion of the arterial wall [60].

A recent ex vivo study explored the effectiveness of PCCT to quantify vulnerable plaque features (e.g., fibrous cap thickness, fibrous cap area, and lipid-rich necrotic core area) and compared PCCT features with histological measurements. PCCT and histological measurement of fibrous cap thickness, fibrous cap area, and lipid-rich necrotic core area did not show significant differences (*p* > 0.05) [61].

### 3.5. Multi-Contrast-Material Applications

This game-changing technology enables different exogenous contrast agents to be discerned and takes full advantage of the capability of K-edge imaging with the introduction of novel contrast agents (e.g., nanoparticles). K-edge imaging enables the recognition of the binding energy between the inner electron shell and the atom as a specific signature, permitting a specific and quantitative evaluation of different contrast agents [18]. These novel contrast agents promise to overcome some limitations of iodine-based agents, including rapid circulation, short retention time, and the similar HU value of iodine to that of calcium at high kilovolt tube voltages [62]. The feasibility of using PCCT to perform multi-contrast imaging of three contrast materials was recently demonstrated in different phantom studies, allowing the creation of a separate material density map for each contrast agent [8,18,22,29,63]. 

A recent in vivo study explored the capability of PCCT to simultaneously discriminate between three contrast agents, namely, intravenous gadolinium and iodine, and oral bismuth, in an animal model, and reported the feasibility of PCCT to differentiate the three K-edge contrast agents in vivo [64].

Recent works have been performed on the use of nanoparticles for PCCT [20,21,22,65,66,67,68]. Si-Mohamed compared PCCT-enabled K-edge imaging in combination with gold nanoparticles with conventional CT images, histologic examination, and transmission electron microscopy data to detect the macrophage burden within rabbit atherosclerotic aortas [22]. A good correlation between the gold concentration and the macrophage area was found (r = 0.82; 95% CI: 0.67, 0.91; *p* = 0.001), highlighting the potential role of PCCT in atherosclerosis in terms of plaque composition and vulnerability [22]. Another in vivo and phantom study demonstrated the potential of PCCT in association with a gold high-density lipoprotein nanoparticle contrast agent to identify macrophage burden, calcifications, and stenosis of atherosclerosis plaques [69]. 

Similarly, tungsten-based and ytterbium-based contrast media have shown to improve atherosclerotic imaging with respect to lumen and plaque visualization [70,71]. Further potential applications of nanoparticles include visualization of tumor vasculature, detection of bleeding, and vascular abnormalities after treatment. Riederer et al. investigated the potential of PCCT to discriminate between liquid embolic agents and iodinated contrast medium using a tantalum-based contrast [72]. The authors demonstrated in a phantom study that PCCT can provide a tantalum density map, differentiating between tantalum and iodine, and enabling the reduction in artifacts due to the liquid embolic agents in patients after vascular occlusion therapy [72]. Gold nanoparticles and liposomal iodine were used in an in vivo study to quantify tumor blood volume and vascular permeability as indicators of cancer angiogenesis [73]. The conjunction of PCCT with gold nanoparticles in the study by Moghiseh et al. allowed the identification and quantification of specific monoclonal antibody-labeled gold nanoparticles with accurate detection of tumor heterogeneity [74].

### 3.6. Myocardial Tissue Imaging

PCCT ensures dual-energy or multi-energy acquisition at a single X-ray tube potential thanks to its energy-discrimination capability. In contrast to DECT, PCCT can differentiate more than two contrast media in each voxel at the time of acquisition. 

This technology can be applied to determine the extent of cardiac damage in myocardial infarction using a double-contrast agent. The first in vivo experiments were conducted in a canine model with myocardial infarction by injecting gadolinium-based and iodine contrast media [27]. The authors demonstrated that these multi-contrast agents can combine first-pass iodine and late gadolinium maps to discriminate between blood pool, scar, and remote myocardium [27]. 

**Figure 7:**
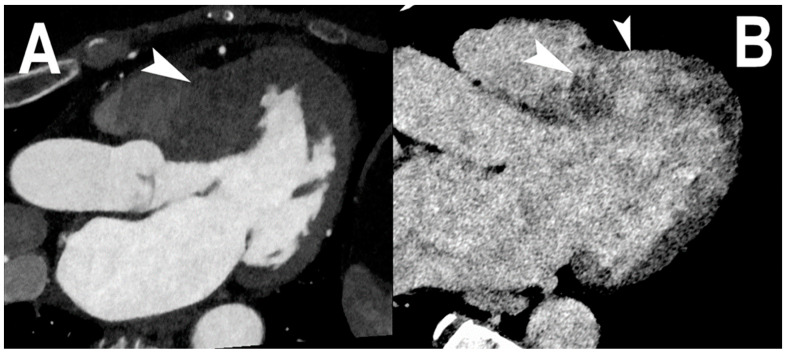
Cardiac PCCT of myocardial late enhancement. In this example (**A**,**B**), a three-chamber projection is shown of a patient with a severe phenotype of hypertrophic cardiomyopathy that affects mostly the mid-basal septum of the left ventricle ((**A**); arrowhead). Seven minutes after the intravenous administration of iodine-based contrast material, the transmural accumulation of iodinated CM in the hypertrophic left ventricular wall, also referred to as late enhancement, is clearly visible ((**B**); arrowhead). This phenomenon is equivalent to the one observed in cardiac magnetic resonance and well established in clinical practice. What is special about this imaging with PCCT is that spectral imaging is contextual with standard cardiac CT protocols and it does not require specific protocols.

Quantification of material concentration may be useful for myocardial perfusion analyses since this allows the exact quantification of the contrast agent in the myocardium [27,28,75]. Notably, iodine maps represent a well-known CT technique to assess myocardial perfusion and have also been validated for the quantification of myocardial late iodine enhancement on DECT [76,77]. The energy threshold capability of PCCT enables minimization of the spectral overlap of DECT, improving the contrast-to-noise ratio and the quantitative capabilities to estimate contrast media concentrations [18]. An example of myocardial late enhancement with PCCT is depicted in Figure 7. PCCT has been demonstrated to provide precise iodine quantification (root mean square error of 0.5 mgI/cc) at different phantom sizes [78].

A recent case report demonstrated the usefulness of spectral CT in clinical practice [11]. Polacin et al. described a case report of a 61-year-old male with acute chest pain who underwent a PCCT scan for suspected acute coronary syndrome. Dual-energy-derived iodine maps from PCCT demonstrated a small ischemic transmural scar, confirmed with late gadolinium cardiac magnetic resonance [11].

Combined iodine/gadolinium injection imaging may be also useful for endovascular leak assessment, as recently demonstrated in a phantom study [79]. Material maps derived from PCCT allowed a reliable distinction of contrast media and aneurysmatic calcifications [79].

An in vivo study explored the radiomics features of the left ventricle myocardium in 30 patients using first-generation, whole-body, dual-source PCCT, reporting an association of coronary artery calcifications and texture analysis [15]. The authors highlighted the potential role of PCCT to overcome the well-known limitation of radiomics analysis in comparison with EID CT, thanks to its higher spatial resolution and contrast-to-noise ratio, and fewer artifacts [15]. Tharmaseelan et al. investigated the texture changes of periaortic adipose tissue in relationship with aortic calcifications using PCCT, demonstrating an association of periaortic adipose tissue with the presence of local aortic calcifications using radiomics analysis [80].

Recently, CT has been demonstrated to be an alternative method to extracellular volume fraction (ECV) quantification in comparison with cardiac magnetic resonance that represents the current non-invasive reference standard. The capability to characterize myocardial tissue using PCCT was emphasized in the in vivo study of Mergen et al. [81]. The authors investigated the feasibility and accuracy of extracellular volume quantification in 30 patients with severe aortic stenosis using PCCT with virtual mono-energetic and dual-energy iodine maps [81]. Virtual mono-energetic and dual energy-derived ECV quantification showed a high correlation (r = 0.87, *p* < 0.001) with narrow limits of agreements and a mean error of 0.9%.

### 3.7. Dose and Contrast Media Reduction

PCCT can boost the attenuation of iodinated contrast media. It is well known that the linear attenuation coefficient of iodine increases with decreasing X-ray energy. This physical aspect results in the possibility of using a lower amount of intravenous contrast media, achieving the same diagnostic results as a full-dose conventional CT examination. This was demonstrated for CCT in a phantom study where the use of virtual mono-energetic image reconstruction at 40 KeV on PCCT allowed reduction in the contrast media concentration by up 50% [82]. Several studies have shown an improved contrast-to-noise ratio using PCCT in comparison with conventional CT scanners, resulting in iodine contrast concentration reduction. Kappler et al. investigated these features in a water phantom, reporting an increased attenuation of iodine with similar image noise compared to conventional CT scanners [83].

An anthropomorphic phantom and ex vivo study investigated the iodine contrast-to-noise ratio in commercial-energy-integrated and PC detectors simulating four patient sizes at four tube potential settings [40]. The authors demonstrated a mean increase in contrast-to-noise ratio of 11%, 23%, 31%, and 38% in comparison with commercially available CT scanners at 80, 100, 120, and 140 kV, respectively [40]. Further, PCCT of a cadaveric human showed decreased artifacts (e.g., beam-hardening and blooming) in the high-energy bin images and improved contrast in low-energy bin images compared to energy-integrated-detector CT [40].

Sawall et al. explored the potential of PCCT to increase iodine contrast, reduce administered radiation dose, or reduce contrast media volume in a phantom of different sizes (small, medium, and large) using an energy-integrated-detector, single-bin PCCT, and two-bin PCCT [25]. The average contrast-to-noise ratio improved using all tube voltages and phantom sizes with an augmentation up to 30% (small: 10%, medium: 18%, large: 30%) with single-bin energy, and up to 37% (small: 13%, medium: 25%, large: 37%) with two-bin energy, highlighting a potential radiation dose reduction of up to 46% [25]. These findings suggested that PCCT can reduce the amount of contrast media required. In this regard, patients allergic to iodine or with renal insufficiency may benefit from spectral CT.

The improvement in electronic noise on image quality by PCCT was assessed for various cardiovascular applications at a low radiation dose. Superior quality of CAC scoring at low radiation doses was described in an in vitro, ex vivo, and in vivo study [38]. The absence of electronic noise in combination with improved soft-tissue contrast allowed the reduction in the radiation dose of CAC scoring [38]. A significant dose reduction (up to 67%) in CAC scoring was also described in an anthropomorphic thorax phantom study using virtual mono-energetic images from PCCT [16].

## 4. Challenges

As for all emerging technologies, there are several technical challenges that must be addressed before the full potential of PCCT can be deployed in clinical practice, including photon flux-independent effects (e.g., charge sharing, charge trapping, and k fluorescence escape) and photon flux-dependent effects (e.g., pulse pile-up) [84,85,86]. Charge sharing occurs when X-ray photons arrive near the boundary between pixels and the cloud is counted in multiple adjacent pixel electrodes [87,88,89,90]. Charge sharing can also occur from the emission of a characteristic photon. Different schemes have been developed to correct for this phenomenon [91,92]. Secondary photons from fluorescence in the detector can be detected in neighboring pixels, causing multiple events that share the total energy of the incident photon. This effect is responsible for the lower limit of the detector pixel size in practical applications [93]. The pulse pile-up is due to the fact that, at very high X-ray flux, the generated voltage pulses overlap in time and are incorrectly counted as a single photon, affecting both energy resolution and image quality [94,95]. The probability of pulse pile-up can be reduced by decreasing the pixel size. However, at realistic CT flux rates, this effect does not play a major role [94].

With increased spatial resolution and multi-energy acquisition, there are also related practical challenges of higher-image data files and an increased burden on data storage, interpretation, and post-processing by human operators [96]. In regard to multi-energy acquisition and K-edge imaging, there are still some issues to overcome before considering a clinical application. These include the gadolinium doses required for in vivo imaging, which are higher in comparison with the current dose needed and recommended in cardiac magnetic resonance, and the fact that other contrast agents (e.g., nanoparticles) are actually still in an experimental stage [24].

However, the major limitations remain the high cost and lack of widespread availability of this technology [96]. Indeed, the elevated costs associated with the production of high-quality PCDs may have a negative impact on the large-scale diffusion of the PCCT technology in the near term. 

## 5. Conclusions

PCCT promises to dramatically change the clinical application of CT in cardiovascular imaging in the coming years.

Further studies are warranted to evaluate the clinical benefits of PCCT for clinical practice in cardiovascular imaging. Specifically, using the data of prospective trials (e.g., Clinical Impact of Cardiac Photon Counting CT [NCT05240807]) may be the first attempt to evaluate the performance of this new CT technology in comparison with conventional CT in applications in the cardiac field. In particular, PCCT will yield clinically important differences that can affect the patient’s management in coronary artery and stent imaging. 

## Figures and Tables

**Table 1:** Benefits of photon-counting detectors and impact on cardiovascular applications.

Benefits of Photon-Counting Detectors	Potential Cardiovascular Applications
Higher spatial resolution	Stent imaging Coronary lumen evaluation Atherosclerotic plaque imaging Coronary artery calcium scoring Aortic valve calcification score
Improved iodine signal	Coronary lumen evaluation Stent imaging
Multi-energy acquisition	Coronary lumen evaluation Atherosclerotic plaque imaging Dose reduction Coronary artery calcium scoring Aortic valve calcification score
Energy binning	Stent imaging Atherosclerotic plaque imaging Dose reduction Myocardial tissue characterization.
Artifact reduction	Coronary lumen evaluation Stent imaging Atherosclerotic plaque imaging

**Table 2:** Studies on PCCT for cardio-vascular applications.

Authors	Years	Model	Number of Patients	Clinical Application	Results
Si-Mohamed et al. [30]	2022	In vivo (human)	14	Coronary lumen	Proportions of score improvement with PCCT images (compared to EID-CT images) for overall quality and diagnostic confidence were 57% (95% CI: 41, 72) and 55% (95% CI: 39, 70), respectively, and 48% (95% CI: 33, 63), 51% (95% CI: 35, 67), and 69 (95% CI: 53, 82) for coronary proximal lumen, coronary distal lumen, and coronary wall, respectively.
Rotzinger et al. [31]	2021	In vitro		Coronary lumen	PCCT provided higher spatial resolution, lower noise magnitude, and superior lipid core detectability than EID-CT.
Koons et al. [12]	2022	In vitro		Coronary lumen	PCCT demonstrated an improvement in plaque/lumen delineation and a more accurate stenosis quantification for all plaques than EID-CT.
Allmendiger et al. [32]	2022	In vitro		Coronary lumen	An image reconstruction algorithm using PCCT decreases blooming artifacts caused by heavily calcified plaques and improves image interpretability.
Si-Mohamed et al. [30]	2022	In vivo (human)	14	Pericoronary fat tissue	Proportion of improvement in diagnostic quality for pericoronary fat tissue of 36% (95% CI, 22–52) in PCCT versus EID-CT.
Rotzinger et al. [31]	2021	In vitro		Atherosclerotic plaque composition	PCCT provided superior detectability for simulated 0.5 mm thick non-calcified plaques (AUC ≈ 95% vs AUC ≈ 75%) and lipid-rich atherosclerotic plaques (AUC = 85% vs. AUC = 77,5%) in comparison with EID-CT.
Si-Mohamed et al. [30]	2022	In vivo (human)	12 (non-calcified plaques) 10 (calcified plaques)	Atherosclerotic plaque composition	Proportions of score improvement with PCCT versus EID-CT images for coronary calcification and noncalcified plaque were 100% and 45% (95% CI: 28, 63), respectively.
Si-Mohamed et al. [22]	2021	In vivo (rabbit)	11	Atherosclerotic plaque composition	A good correlation between the gold concentration and the macrophage area was found (r = 0.82; 95% CI: 0.67, 0.91; *p* = 0.001).
Boussel et al. [9]	2014	In vitro	3	Atherosclerotic plaque composition	PCCT demonstrated to identify plaque components by measuring differences in contrast agent concentration and spectra attenuation.
Boccalini et al. [33]	2022	In vivo (human)	8	Coronary stent Dose reduction	Superior stent and lumen visibility with fewer blooming artifacts and lower dose radiation for PCCT versus conventional CT.
Mannil et al. [23]	2018	In vitro		Coronary stent	In comparison with EID-CT, PCCT offered superior in stent visibility, fewer blooming and partial volume artifacts, with a lower increase in the attenuation of the lumen inside the stent.
Symons et al. [34]	2018	In vitro		Coronary stent	Better luminal depiction with lower image noise in PCCT compared to EID-CT.
Harmel et al. [35]	2021	In vivo (human)	5	Aortic calcium score	Excellent correlation of aortic valve calcium score and volumes between virtual non-contrast and true-contrast images (r = 0.945, *p* = 0.01; r = 0.938, *p* = 0.01; respectively).
Van Der Werf et al. [36]	2022	In vitro		Coronary artery calcium score	A comparable CAC score for routine clinical protocol between PCCT and conventional CT was demonstrated. Further, PCCT showed increased detectability and accuracy in CAC at reduced slice thickness.
Eberhard et al. [37]	2022	In vitro In vivo	20	Coronary artery calcium score	PCCT showed decreasing CAC score at increasing iterative image reconstruction algorithm levels (*p* < 0.001) and increasing keV levels (*p* < 0.001).
Skoog et al. [10]	2022	In vitro		Coronary artery calcium score	High correlation and agreement were observed between the CAC score derived from PCCT and EID-CT.
Symons et al. [38]	2019	In vitro In vivo	10	Coronary artery calcium score Dose reduction	In vitro: CAC score reproducibility was significantly higher for the PCCT at the lowest dose setting (50 mAs) (*p* = 0.002). In vivo: agreement between standard-dose and low-dose CAC score was significantly better for the PCCT than for the conventional CT.
Symons et al. [27]	2017	In vivo (canine)	3	Myocardial tissue characterization	The distribution and morphology of myocardial scar by PCCT single-energy and gadolinium map correlated well with MRI and histology. The gadolinium map had 31% higher contrast-to-noise ratio than the CT single-energy images for identifying infarct versus remote tissue.
Mergen et al. [39]	2022	In vivo (human)	30	Myocardial tissue characterization	With PCCT virtual mono-energetic and dual-energy-derived ECV quantification showed high correlation (r = 0.87, *p* < 0.001) with narrow limits of agreements and a mean error of 0.9%.
Van Der Werf et al. [16]	2021	In vitro		Dose reduction	Virtual mono-energetic images from PCCT demonstrated a dose reduction up to 67%.
Gutjahr et al. [40]	2016	In vitro Ex vivo		Contrast-to-noise ratio	In vitro: a mean increase in contrast-to-noise ratio of 11%, 23%, 31%, 38% was demonstrated for PCCT in comparison with commercially available CT scanners at 80, 100, 120, and 140 kV, respectively. Ex vivo: Compared to EID-CT, PCCT allowed for decreased artifacts (e.g., beam-hardening and blooming) in the high-energy bin images and improved contrast in low-energy bin images.

## Data Availability

Not applicable.
